# Gynecologic Malignancies Post-LeFort Colpocleisis

**DOI:** 10.1155/2014/846745

**Published:** 2014-12-01

**Authors:** Rayan Elkattah, Alicia Brooks, R. Keith Huffaker

**Affiliations:** ^1^Department of Obstetrics and Gynecology, Quillen College of Medicine, East Tennessee State University, 325 N State of Franklin Road, Johnson City, TN 37604, USA; ^2^Quillen College of Medicine, East Tennessee State University, Johnson City, TN 37614, USA; ^3^Division of Female Pelvic Medicine and Reconstructive Surgery, Department of Obstetrics and Gynecology,, Quillen College of Medicine, East Tennessee State University, 325 N State of Franklin Road, Johnson City, TN 37604, USA

## Abstract

*Introduction*. LeFort colpocleisis (LFC) is a safe and effective obliterative surgical option for older women with advanced pelvic organ prolapse who no longer desire coital activity. A major disadvantage is the limited ability to evaluate for post-LFC gynecologic malignancies. *Methods*. We present the first case of endometrioid ovarian cancer diagnosed after LFC and review all reported gynecologic malignancies post-LFC in the English medical literature. *Results*. This is the second reported ovarian cancer post-LFC and the first of the endometrioid subtype. A total of nine other gynecologic malignancies post-LFC have been reported in the English medical literature. *Conclusions*. Gynecologic malignancies post-LFC are rare. We propose a simple 3-step strategy in evaluating post-LFC malignancies.

## 1. Introduction

Symptomatic pelvic organ prolapse affects millions of women. Approximately 200,000 surgeries for POP are performed in the United States annually [1] and represent either reconstructive or obliterative procedures. Colpocleisis is an obliterative procedure which is an effective and minimally invasive option for women who cannot tolerate or do not desire extensive reconstructive surgery and who do not desire future vaginal intercourse. The advantages offered by colpocleisis include shorter operative time, decreased perioperative morbidity, and a low risk of pelvic organ prolapse recurrence. Besides precluding vaginal intercourse, another major disadvantage of colpocleisis is the limited ability to evaluate the cervix, uterus, or ovaries through a vaginal route postoperatively, which at times may delay the diagnosis of gynecologic malignancies. We present the first reported case of endometrioid ovarian cancer diagnosed after a LeFort colpocleisis (LFC) and review the reported literature pertaining to gynecologic malignancies post-LFC.

## 2. Methods

### 2.1. Case Illustration

A previously healthy nulligravid and menopausal 76-year-old female presented with worsening pelvic organ prolapse (POP) symptoms. Her medical and surgical histories were significant for left shoulder and right knee surgeries. She denied any history of abnormal Papanicolaou tests and sexually transmitted illnesses. Besides obesity, she denied having any medical problems. Her body mass index was 35 kg/m^2^. She reported a family history of breast cancer in her maternal aunt but denied any other personal or family history of any other malignancies. Her medications included daily low-dose acetylsalicylic acid and a calcium supplement. She denied being on any hormonal replacement therapy. Recent colonoscopy, mammogram, and Papanicolaou test were normal. She denied smoking, alcohol use, and use of illicit drugs. Urogynecologic evaluation using the Pelvic Organ Prolapse Quantification (POP-Q) system revealed stage 4 uterovaginal prolapse. The patient also had postmenopausal atrophic vaginitis. There were no palpable masses. In favor of a definitive and long-lasting treatment for her POP, she was counseled about vaginal reconstructive and obliterative surgery options. Uterine and ovarian conservation were requested by the patient following normal clinical examination. She underwent LeFort colpocleisis, levator plication, perineorrhaphy, and cystourethroscopy. She had a quick recovery and was doing well at her 8-week postoperative visit with no recurrent prolapse. She presented 6 months later with painless vaginal spotting with no other associated symptoms. Atrophic vulvovaginitis was suspected as a cause of this spotting and the patient was treated with topical vaginal conjugated equine estrogen. Her bleeding persisted despite a month of therapy. At this point, further evaluation included abdominal and rectal exams that were normal. Transvaginal ultrasonography was not feasible with an obliterated vagina. An abdominal ultrasound was limited by body habitus but showed a questionable 4 × 4 cm pelvic fluid collection with no particular pattern on Doppler mode and was thought to resemble a hematoma. Endometrial thickness was 5 mm. In light of this undiagnosed vaginal bleeding, the suspected pelvic fluid collection and thickened endometrium the patient was counseled about the differential diagnosis of vaginal bleeding including atrophic vaginitis and other gynecologic sources, namely, cervical and endometrial pathology and she was informed of the need to further evaluate her bleeding. We offered the patient the option of colpocleisis “reversal” by taking the repair down and reaccessing the cervix for a proper endometrial evaluation but she had refused given the excellent prolapse correction. Furthermore, our experience in accessing the cervix and endometrium through the lateral vaginal canals is minimal and thus the decision to perform a total abdominal hysterectomy (TAH) to evaluate the endometrial lining and the questionable fluid collection was taken. During the TAH, an 8 cm × 5 cm × 5 cm left ovarian irregular mass was identified. Initial visual and tactile evaluation of the pelvic and abdominal structures was normal except for cecal and posterior vaginal nodules. Intraoperative gynecologic oncology consultation was obtained. A frozen section of the left ovary revealed adenocarcinoma. Staging then followed. Pelvic washings were obtained upon initial abdominal entry. Uterus, fallopian tubes, right ovary, and omentum were resected. Peritoneal biopsies were also obtained. All these specimens were negative for metastatic disease. Resected cecal and posterior vaginal nodules were positive for metastatic adenocarcinoma. She was diagnosed with stage IIIB grade 1 endometrioid ovarian cancer.

The patient underwent adjuvant chemotherapy with paclitaxel and carboplatin thereafter. She tolerated the treatments well and was doing well at her 3-month postchemotherapy follow up. Her CA-125 dropped from 25 u/mL to 6 u/mL at the time of initial diagnosis and by her last chemotherapy session, respectively. Both limited vaginal exam and digital rectal exam were normal. Chest X-ray and abdominal/pelvic computed tomography scan were unremarkable. She remains in remission 2 years after chemotherapy and regularly follows up with the gynecologic oncologist.

### 2.2. Literature Review of Gynecologic Malignancies Post-LFC

Although the development of gynecologic malignancies post-LFC is rare, it is more than a theoretical risk [2]. We reviewed the Pubmed/Medline database and open-sources using the following search terms: “partial/complete/LeFort colpocleisis” and “ovarian,” “endometrial/uterine,” “cervical,” “fallopian/tubal,” “vaginal,” and “malignancy/carcinoma/cancer/neoplasia.” Our comprehensive English language literature review yielded a total of nine reported post-LFC gynecologic malignancies since 1948 [2–8]. These comprise five endometrial cancers, individual cases of vaginal, cervical, and ovarian cancer in addition to an unspecified gynecologic malignancy [6]. Hanson and Keettel reported that prior to 1936, only one case report of malignancy developing after a Le Fort operation had appeared in the literature but no specific type was mentioned. We also retrieved referenced papers from within the articles we referenced in our paper all the way back to 1936 in an attempt to enhance the capture of any gynecologic malignancy reported in the English medical and gynecologic literature.  Table 1 summarizes the reported malignancies. To our knowledge, this is the second reported post-LFC ovarian malignancy and the first describing the endometrioid type. The only other ovarian malignancy reported was in 1975 by Sudo et al. [7]. They reported a case of postmenopausal intermittent vaginal bleeding in a 56-year-old woman three years after LFC. Her Papanicolaou test showed mild atrophic atypia and she thereafter underwent a fractional dilation and curettage through the right egress channel that was accessed after sequential dilation using Hegar dilators and scalpel dissection of fibrous bands. The initial pathology was suggestive of moderately differentiated adenocarcinoma. She subsequently underwent laparotomy and was found to have a 5 × 5 cm left adnexal mass with papillary seeding over the fallopian tubes, uterus, right ovary, and diffuse tumor seeding over the small bowel and omentum. Cytoreductive surgery followed and the final pathology reported a papillary adenocarcinoma of the left ovary. She also underwent radiation therapy.

## 3. Discussion

Colpocleisis is a safe and effective obliterative surgical option for older women with advanced pelvic organ prolapse (POP) who no longer desire coital activity [3]. Compared to reconstructive vaginal surgery in elderly women with multiple medical comorbidities, colpocleisis offers several advantages: simplicity, reported good outcomes, decreased period of anesthesia, shorter operative time, less blood loss [9], and high patient satisfaction [10]. In a LeFort colpocleisis, equivalent areas of the anterior and posterior vaginal epithelium are removed before the remaining lateral vaginal epithelium is approximated anterior to posterior with a series of interrupted absorbable sutures to create egress drainage channels. The pubocervical connective tissues anteriorly and the rectovaginal connective tissues posteriorly are progressively reduced into the pelvis with a series of anterior to posterior inverting absorbable sutures reducing the cervix, uterus, and other prolapsed structures back into the pelvis. The residual vaginal epithelium is approximated over the connective tissue and a perineorrhaphy and/or levator myorrhaphy usually conclude the surgery. A major disadvantage of this procedure is the postoperative compromised facility for proper evaluation of gynecologic pathology [2], particularly malignancies. Egress channels provide an outlet for any drainage and may allow for a timely diagnosis of several malignancies, particularly when associated with persistent vaginal bleeding or discharge. Vaginal bleeding post-LFC is uncommon and the broad differential diagnosis includes postmenopausal atrophic changes, cervical, vaginal, and endometrial pathology. Since there are no determined associations between LFC and increased risks of gynecologic malignancies, we do believe that there are likely several other ovarian cancers that have developed post-LFC; however, none have been published and reported. Furthermore, the paucity of reported malignancies may be explained by the fact that a transvaginal hysterectomy with a bilateral salpingooophorectomy is performed at the time of a colpocleisis as a means to eliminate the risk of upper gynecologic pathology, particularly malignancies.

### 3.1. Pre-LFC Evaluation

Due to the obliterative nature of LFC, postoperative gynecologic evaluation is limited. This reflects the importance of preoperative assessment and counseling. Patients should be made aware of the potential delay and limitation in diagnosing gynecologic pathology post-LFC. The precolpocleisis exam should include a careful speculum exam with visual inspection of the cervix and vagina followed by palpation of the entire vagina [8] as well as a bimanual and rectal exam in addition to cervical cytology, transvaginal ultrasound, and endometrial sampling when indicated [11]. The role of transvaginal ultrasound as a preoperative tool in the assessment of gynecologic pathology has been suggested by several authors [11, 12]. There is a 1.1% risk of missing an early endometrial carcinoma in postmenopausal women when preserving the uterus for prolapse surgery and thus a preoperative ultrasound is recommended in all cases followed by endometrial sampling when indicated [11]. A recent report by Frick et al. showed a 2.6% risk of unexpected abnormal uterine pathology (endometrial hyperplasia or carcinoma) in postmenopausal women without uterine bleeding [13]. They also recommended against uterine preservation at the time of prolapse surgery, particularly in women with postmenopausal bleeding and negative endometrial evaluation [13]. Despite this data, there is no consensus among gynecologists about the necessity for a transvaginal ultrasound and/or endometrial sampling in the preoperative evaluation of colpocleisis [3], especially when a patient is otherwise asymptomatic. From a cost-utility standpoint, Kandadai et al. argued against performing an endometrial evaluation before LFC in low-risk women as that strategy seems superior to endometrial biopsy and ultrasound [14]. In light of this conflicting data, it seems prudent that a gynecologic evaluation may reduce the risk of unexpected uterine pathology [13], particularly before prolapse surgery but is likely not cost-effective. With respect to ovarian cancer, the role of preoperative screening with transvaginal ultrasound, tumor markers, and/or bimanual examination in asymptomatic women remains unclear as it does not lead to reduced mortality and is associated with unnecessary surgery [15]. In a study by Bonnar et al., incidental unsuspected ovarian neoplasms were discovered in 13 of 500 patients (2.6%) undergoing vaginal hysterectomy for uterovaginal prolapse [16]. Our patient's ovarian cancer was likely present and missed on initial evaluation and limited abdominal ultrasound. On the other hand, a recent study determined that 39% of low-risk patients undergoing LFC had unnecessary pre-/intraoperative diagnostic testing, and that a negligible incidence of malignancy was revealed [17].

### 3.2. Post-LFC Evaluation

The postcolpocleisis gynecologic exam includes an evaluation of the external genitalia in addition to a limited vaginal and complete rectal exam. There are currently no practice guidelines for the evaluation of gynecologic malignancies post-LFC. In light of the limited literature available, we compile a simple 3-step approach in evaluating post-LFC malignancies: (1) routine gynecologic exams; (2) cytology/biopsy; and (3) an imaging study. This 3-step approach should be used in any suspected gynecologic malignancy post-LFC, and not with suspected endometrial pathology only. As in prior reported cases, sending a sample of the drainage for cytology may be diagnostic particularly with vaginal and cervical lesions [2, 8]. Imaging is also extremely helpful as an adjunct to the gynecologic exam in persistent post-LFC vaginal discharge and/or bleeding. Transabdominal or transperineal ultrasonography may be utilized as an initial imaging modality. Other modalities include magnetic resonance imaging and/or computed tomography (CT). CT scan or, when possible, a biopsy is recommended to assess persistent vaginal discharge or pelvic pain presenting remote from obliterative surgery [8]. With postmenopausal bleeding, endometrial evaluation with ultrasound should be done and if warranted, an endometrial sample may be obtained via rigid hysteroscopy [3] or by dilating the egress channels to allow for pipelle insertion or a dilation and curettage. Until an accurate screening test for ovarian cancer is developed, it is unlikely that routine ultrasounds and tumor markers will be of any survival benefit particularly post-LFC.

## 4. Conclusion

LeFort colpocleisis is a safe and effective obliterative surgical option for older women with advanced pelvic organ prolapse who no longer desire coital activity. Significant tissue pathology is not anticipated; however, many patients with prolapse are elderly and are at a risk of developing a gynecologic malignancy [18]. Commonly, low risk patients undergoing LFC have unnecessary testing, which may draw more scrutiny in an increasingly cost conscious medical environment [14, 17]. This case highlights the clinical dilemma faced in both the preoperative work-up of the low risk patient and the challenges of subsequent evaluation of a post-LFC patient with symptoms suspicious for gynecologic malignancy. We believe that the role of pre-LFC evaluation is still crucial in identifying gynecologic pathology prior to surgery but this should be tailored to each patient. Compliance to close and prompt follow-up is highly advised as well. Assuming a normal work-up was undertaken preoperatively, the objective of our report is to describe the steps that should be taken when a post-LFC gynecologic malignancy is suspected and is based on the post-LFC gynecologic malignancy literature published thus far. Accordingly, a 3-step strategy in evaluating post-LFC malignancies may prove useful in the evaluation of gynecologic malignancies in patients who underwent a LeFort colpocleisis.

## Figures and Tables

**Table 1:** Gynecologic malignancies post-LeFort colpocleisis.

Author [reference]	Year	Diagnosed malignancy	Cases within a LFC series or reported individually	Age at diagnosis (years)	Presentation	Approximate interval between LFC and malignancy diagnosis	Diagnostic modality	Treatment
Mazer and Israel [4]	1948	Endometrial adenocarcinoma	1 in 43	62	Vaginal bleeding	9 years	TAH BSO	Unspecified radiation therapy

Falk and Kaufman [5]	1955	Endometrial adenocarcinoma	1 in 100	Unspecified	Vaginal bleeding	Unspecified	TAH	None

Hanson and Keettel [6]	1969	Endometrial adenocarcinoma	1 in 288	Unspecified	Vaginal bleeding	3 years	Colpocleisis take down, D & C	ICR, TAH BSO
		Endometrial adenocarcinoma	1	91	Vaginal bleeding	16 years	D & C	ICR
		Unspecified^*^	1	Unspecified	Unspecified	Unspecified	Unspecified	Unspecified

Sudo et al., [7]	1976	Ovarian: papillary adenocarcinoma	1	56	Vaginal bleeding	3 years	Dilation of lateral channel, D & C	TAH BSO, external pelvic radiotherapy

Yamakawa et al., [2]	1998	Cervical: squamous cell carcinoma	1	89	Purulent vaginal discharge and bleeding	7 years	Papanicolaou test, abdominal/pelvic CT	None (patient deceased prior to treatment)

Cho et al., [8]	2011	Vaginal: squamous cell carcinoma	1	75	Recurrent purulent vaginal discharge and perianal pain	1 year, 4 months	Pelvic CT, Biopsy	External pelvic radiotherapy

Harmanli et al., [3]	2013	Endometrial: clear cell carcinoma	1	74	Vaginal bleeding	1 year, 2 months	Transvaginal-channel hysteroscopy, D & C	Robotic TLH BSO, lymphadenectomy

Total = 9								

^*^Per Hanson and Keettel [6] prior to 1936, only 1 case report of malignancy developing after a LeFort operation had appeared in the literature. The type of cancer was not specified, however.

Legend: TAH: total abdominal hysterectomy; TLH: total laparoscopic hysterectomy; BSO: bilateral salpingooophorectomy; D & C: dilation and curettage; ICR: intracavitary radium; CT: computed tomography.
